# Prevalence of the SigB-Deficient Phenotype among Clinical *Staphylococcus aureus* Isolates Linked to Bovine Mastitis

**DOI:** 10.3390/antibiotics12040699

**Published:** 2023-04-03

**Authors:** Anna Walzl, Helene Marbach, Darya Belikova, Claus Vogl, Monika Ehling-Schulz, Simon Heilbronner, Tom Grunert

**Affiliations:** 1 Institute of Microbiology, Department of Pathobiology, University of Veterinary Medicine, A-1210 Vienna, Austria; 2 Interfaculty Institute of Microbiology and Infection Medicine, University of Tübingen, D-72076 Tübingen, Germany; 3 Molecular Genetics, Institute of Animal Breeding and Genetics, Department of Biomedical Sciences, University of Veterinary Medicine, A-1210 Vienna, Austria

**Keywords:** *Staphylococcus aureus* bovine mastitis, SigB deficiency, *rsbU*, pigmentation, staphyloxanthin, α-hemolysin, proteolysis, persistence

## Abstract

Phenotypic adaptation has been associated with persistent, therapy-resistant *Staphylococcus aureus* infections. Recently, we described within-host evolution towards a Sigma factor B (SigB)-deficient phenotype in a non-human host, a naturally infected dairy cow with chronic, persistent mastitis. However, to our knowledge, the prevalence of SigB deficiency among clinical *S. aureus* isolates remains unknown. In this study, we screened a collection of bovine mastitis isolates for phenotypic traits typical for SigB deficiency: decreased carotenoid pigmentation, increased proteolysis, secretion of α-hemolysin and exoproteins. Overall, 8 out of 77 (10.4%) isolates of our bovine mastitis collection exhibited the SigB-deficient phenotype. These isolates were assigned to various clonal complexes (CC8, CC9, CC97, CC151, CC3666). We further demonstrated a strong positive correlation between *asp23*-expression (a marker of SigB activity) and carotenoid pigmentation (r = 0.6359, *p* = 0.0008), underlining the role of pigmentation as a valuable predictor of the functional status of SigB. Sequencing of the *sigB* operon (*mazEF-rsbUVW-sigB*) indicated the phosphatase domain of the RsbU protein as a primary target of mutations leading to SigB deficiency. Indeed, by exchanging single nucleotides in *rsbU*, we could either induce SigB deficiency or restore the SigB phenotype, demonstrating the pivotal role of RsbU for SigB functionality. The data presented highlight the clinical relevance of SigB deficiency, and future studies are needed to exploit its role in staphylococcal infections.

## 1. Introduction

Chronic, persistent *Staphylococcus aureus* infections are difficult to treat with antibiotics and often recur. Although the role of *S. aureus* virulence factors in acute infections is well established, bacterial factors contributing to persistence are far less understood. The combined effects of insufficient host clearance mechanisms, bacterial immune evasion and ineffective antibiotic therapies are thought to allow the pathogen to survive for prolonged periods in the host [1]. In particular, intracellular survival by switching to the small-colony variant (SCV) phenotype is suggested to be a common reservoir for persistent infections in humans and bovines [2,3]. This mechanism of intracellular survival and SCV formation requires the upregulation of Sigma factor B (SigB), a major stress regulator in *S. aureus* [4]. However, frequent isolation of SigB-deficient strains during *S. aureus* infections suggests an advantage of this phenotype in certain niches [5,6,7]. We recently hypothesized that strains lacking SigB expression might better adapt to the extracellular niche, enabling long-term persistence in chronic mastitis [8]. SigB deficiency is typically associated with high production of proteases and toxins [6,9], suggesting that in bovine mastitis, elevated proteolysis may provide new sources of nutrients, promoting survival in the bovine mammary gland [8]. Moreover, the increased secretion of α-hemolysin (α-toxin, Hla) associated with a more cytotoxic phenotype may improve the capacity to penetrate and disseminate the udder tissue, thus supporting adaptation to the extracellular microenvironment in the mammary gland [10].

The *S. aureus* genome encodes several known sigma factors with different promotor specificities: the housekeeping sigma factor A (SigA) and at least three alternative sigma factors SigB, SigH and SigS [11]. Sigma factors are necessary to initiate transcription, as they bind to the RNA polymerase holoenzyme, guiding it to the promoters of the target gene. In *S. aureus*, the sigma factor SigB is a major stress regulator known to respond to changing environmental conditions, as it can be activated by alkaline shock, salt stress and temperature shift and is induced during the stationary phase [11]. SigB transcription is regulated by the hexa-cistronic *sigB* operon: *mazE-mazF-rsbU-rsbV-rsbW-sigB* [12]. SigA drives the transcription of the whole *sigB* operon, incorporating a *SigB*-dependent positive-feedback loop that leads to the transcription of *rsbVW*-*sigB*. Moreover, the *sigA*-dependent promoter P_mazE_ was demonstrated to contribute to SigB activity by producing a *mazEF*-*rsbUVW*-*sigB* mRNA [13,14]. At the posttranslational level, multiple protein-protein interactions, including a partner-switching mechanism, regulate SigB activity in *S. aureus*. The anti-sigma factor RsbW holds SigB in an inactive complex, whereas the anti-anti-sigma factor RsbV antagonises RsbW, releasing SigB. The released sigma factor can subsequently interact with RNA polymerase and promote gene transcription. RsbV can only act as an anti-anti-sigma factor when non-phosphorylated, and phosphorylation is regulated by the phosphatase RsbU. In addition, RsbU is involved in passing stress stimuli to SigB and, in contrast to *Bacillus subtilis*, might be consistently active at a high level in *S. aureus* without any stimulation [15,16].

SigB regulates the expression of more than 200 genes that control, amongst others, cell-surface and secreted virulence factors such as pigments, toxins and proteolytic enzymes [17]. The golden (“aurum”)-coloured surface pigment results from triterpenoid carotenoids, which act as antioxidants and neutralize reactive oxygen species (ROS), promoting intracellular survival in phagocytes. By interfering with neutrophil killing, they likely promote virulence [18,19]. Staphyloxanthin (STX) is the primary pigment of *S. aureus* produced by the biosynthetic operon *crtOPQMN* [20,21]. While SigB directly binds to the *crtOPQMN* operon for pigment production, the SigB regulon is also linked to multiple global regulatory networks, most notably the accessory gene regulator (*agr*) quorum-sensing system with its effector molecule RNA III and the transcriptional regulator staphylococcus accessory regulator A (SarA) [22,23]. SigB induces the expression of SarA and inhibits the expression of *agr*/RNAIII, thereby suppressing the secretion of toxins, incl. α-hemolysin and staphylococcal proteases [17,24]. SigB deficiency has been shown to be associated with genetic alterations within the *sigB*-locus, resulting in higher RNA III and lower SarA levels. SigB-deficient strains are also reported to secrete higher amounts of proteins often associated with a typical exoprotein pattern [5,8,25]. Collectively, the following phenotypic traits were described as typical for SigB deficiency: (i) reduced pigmentation (white or grey colony colour), (ii) increased proteolysis, (iii) increased α-hemolysin secretion, and (iv) increased protein secretion [5,6,8,9,25]. Since the expression of the alkaline shock protein 23 (Asp23) is solely SigB-dependent, it is often used as an indicator of SigB activity at the transcriptional level [9,26].

Recently, we described the first within-host evolution towards a SigB-deficient phenotype in a naturally infected dairy cow with chronic, persistent mastitis [8]. We demonstrated that a dominant initial variant (IN) was replaced by a host-adapted, SigB-deficient variant (HA), which carried a single nucleotide polymorphism (SNP) in *rsbU*(G368A) within the *sigB*-locus. We assumed that the resulting glycine/aspartate substitution (G122D) within the C-terminal phosphatase domain causes a loss in the RsbV-P-specific phosphatase activity of RsbU, rendering SigB dysfunctional. In the present study, we aimed to determine the prevalence of SigB deficiency among *S. aureus* bovine mastitis isolates, which is, to our knowledge, unknown so far. Here, we employed several phenotypic assays to search for isolates presenting the SigB-deficient phenotype and evaluated the functional status of SigB by *asp23*-transcriptional expression. To determine the genetic basis of SigB deficiency, we further constructed isogenic mutants introducing or reverting the SigB-deficient phenotype.

## 2. Results

### 2.1. Screening for the SigB-Deficient Phenotype in Bovine Mastitis Isolates

#### 2.1.1. Measurement of Extracted Pigments

**Figure 1:**
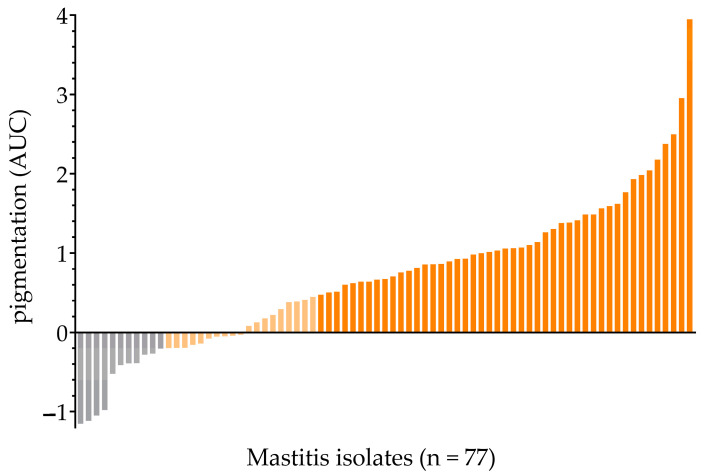
Mastitis isolates were ordered by increased pigmentation (AUC value). (i) non-pigmented (grey), (ii) intermediate-pigmented (light-yellow) and (iii) fully pigmented (orange).

The spectra of SigB-functional reference strains (SH1000, 6850 and IN) showed a characteristic spectral profile of carotenoid pigmentation with peaks at ~440 nm and ~470 nm [27]. Those peaks were absent in the SigB-deficient reference strains (SH1000Δ*sigB*, 8325-4, 6850Δ*sigB* and HA). The baseline was adjusted as a straight line through the values at 390 and 520 nm to determine carotenoid pigment production (Supplementary Figure S1). Three standard deviations (SD) above the mean area under the curve (AUC) of the SigB-deficient reference strains (SH1000Δ*sigB*, 6850Δ*sigB*, 8325-4 and HA) served as a cut-off value (AUC_c_ = −0.2048) for strains not producing pigments. Pigment production of the 77 mastitis-derived isolates revealed AUC values ranging from −1.150 to 3.947, with a mean value of 0.6998 (Figure 1). Isolates were differentiated into non-pigmented (14.3%, 11/77), intermediate-pigmented (24.7%, 19/77), and fully pigmented strains (61.0%, 47/77). Fully pigmented isolates were considered to have fully functional SigB activity and were excluded from further analyses searching for SigB deficiency.

#### 2.1.2. Proteolytic Activity, Immune-Based Detection of α-Hemolysin Secretion and Protein Secretion Profiling

**Figure 2:**
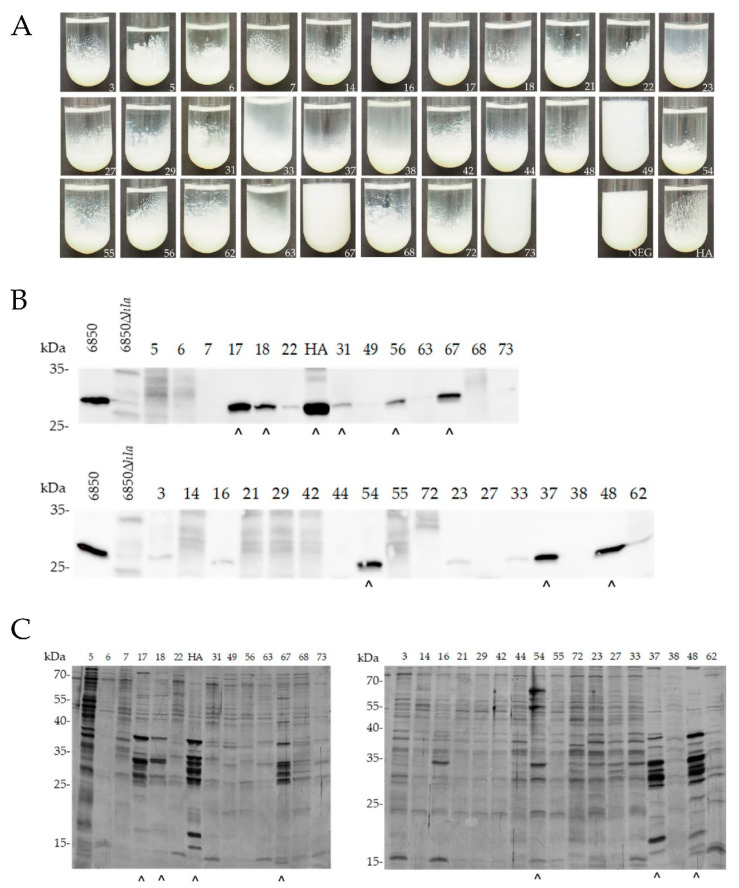
Phenotypic screening of SigB deficiency of non-/intermediate-pigmented mastitis-derived isolates (n = 30). (**A**) Proteolysis in skim milk. Isolates were grown in 1% skim milk and proteolysis was assessed after 24 h. (**B**,**C**) Western blot against Hla (**B**) and SDS-PAGE of bacterial secretion pattern (**C**) were performed from supernatants derived from equal numbers of *S. aureus* cells harvested at OD_600_ = 5. HA, reference strain; NEG, negative control. (^) Strains, positive for Hla secretion and increased protein secretion.

Most non- and intermediate-pigmented mastitis isolates (27/30) showed proteolytic activity, except isolates ID49, ID67 and ID73 (Figure 2A). Eight out of 30 isolates secreted variable amounts of α-hemolysin: ID17, ID18, ID31, ID37, ID48, ID54, ID56 and ID67 (Figure 2B). When comparing silver-stained protein band patterns, we detected increased protein secretion specific for the SigB-deficient phenotype for ID17, ID18, ID37, ID48, ID54 and ID67 (Figure 2C). Interestingly, although isolate ID67 was not proteolytically active, it secreted high levels of α-hemolysin and exoproteins, suggesting a SigB-deficient phenotype.

Overall, based on the phenotypic assays, the following eight isolates (n = 8/77; 10.4%) were considered SigB-deficient: ID17, ID18, ID31, ID37, ID48, ID54, ID56 and ID67 (Table 1). Notably, these isolates were distributed across various clonal complexes (CC8, CC9, CC97, CC151, CC3666).

#### 2.1.3. *Asp23*-Transcriptional Expression (RT-qPCR)

Since SigB positively controls *asp23* transcription, we expected low levels of *asp23* expression (reverse transcription-quantitative polymerase chain reaction, RT-qPCR) for all the SigB-deficient phenotypes (n = 8). We detected an average *asp23* mRNA expression level relative to housekeeping genes of 69.8, ranging from 1.0 to 176.0, while the SigB-functional reference strain SH1000 had a relative expression of 266.0 (Supplementary Figure S2). Most relative *asp23* mRNA expression levels were below 100, indicating minor SigB activity. Interestingly, relative expression levels between 100 and 200 were found for two isolates (ID17 and ID67), indicating moderate SigB activity.

### 2.2. Positive Correlation between asp23-Transcriptional Expression and Pigmentation

**Figure 3:**
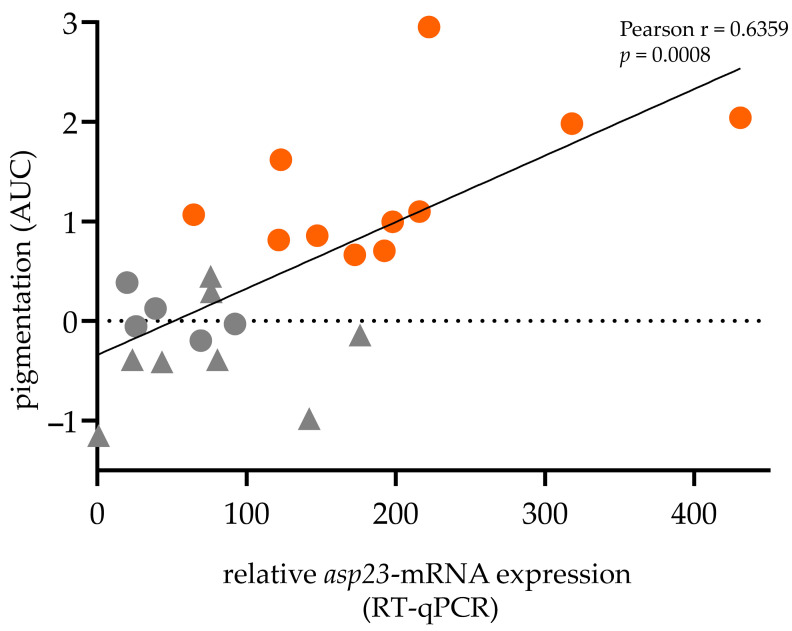
*Asp23*-transcriptional expression positively correlates with staphylococcal pigment production. Pearson correlation coefficient (Pearson r), correlation *p*-value, and best-fit line (linear) shown. Orange: Fully pigmented isolates (n = 11), which are considered SigB-functional; Grey: Non- and intermediate-pigmented isolates (n = 13) including isolates presenting the SigB-deficient phenotype (n = 8; Triangle).

As *asp23* gene transcription indicates the functional status of SigB [9], it was interesting to establish whether *asp23*-transcriptional expression (RT-qPCR) levels correlate with staphylococcal pigmentation (extracted pigments, AUC). Pearson correlation analysis revealed a strong positive correlation between *asp23*-expression and carotenoid pigmentation (r = 0.6359, *p* = 0.0008; Figure 3). Notably, many isolates (5/11) were not assigned to the SigB-deficient phenotype because no increased secretion of α-hemolysin and exoproteins could be detected, although they expressed low levels of *asp23*-mRNA and pigments and showed proteolytic activity.

### 2.3. Sequence Analysis of the sigB Operon and Construction of Mutants to Confirm the SigB-Deficient Phenotype

To search for the genetic basis for SigB dysfunction, we sequenced the *sigB* operon (*mazEF*-*rsbUVW*-*sigB*) of all isolates exhibiting the SigB-deficient phenotype (n = 8). We did not identify substitution mutations or insertion/deletion mutations creating premature stops of translation. However, we have determined several synonymous and non-synonymous nucleotide substitutions by comparing the sequences of the *sigB* operon with known SigB-functional strains (Table 1). In particular, we noticed changes in the *rsbU* gene. We identified the SNP *rsbU*(G395A → S132N) in strain ID31 and *rsbU*(G431T → G144V) in strain ID56, which both were located within the phosphatase domain that has been shown to cause SigB dysfunction when inactivated [9]. For isolate ID18, we obtained three synonymous nucleotide substitutions in *mazF*, *rsbW* and *sigB*, and one SNP in the non-coding region between the genes *rsbV* and *rsbU*, the latter known to bind the feed-forward *sigB*-dependent P_B_ promoter [13,14]. However, for the isolates ID17, ID37, ID48, ID54 and ID67, we could not track possible genetic differences in the *sigB* operon, although presenting the SigB-deficient phenotype.

**Figure 4:**
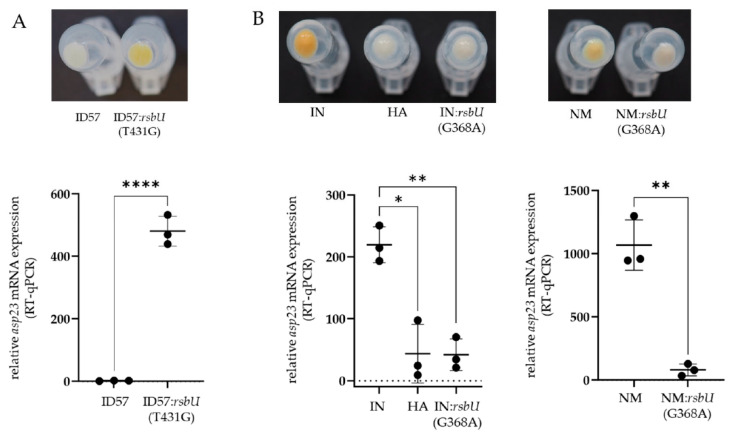
Proof of the causal relationship of SigB deficiency based on a single nucleotide mutation in *rsbU.* Changes in bacterial surface pigmentation and *asp23*-mRNA expression (RT-qPCR) were shown, which are indicators of SigB activity. (**A**) Restoring the SigB-functional phenotype by exchanging the single nucleotide *rsbU*(T431G) in the SigB-deficient bovine mastitis isolate ID57. (**B**) Induction of the SigB-deficient phenotype by exchanging the single nucleotide *rsbU*(G368A) in the SigB-functional initial (IN) bovine mastitis isolate and the SigB-functional human reference strain Newman (NM); (* *p* < 0.05, ** *p* < 0.01, **** *p* < 0.001).

We further constructed isogenic mutants introducing or reverting the SigB-deficient phenotype, serving as a proof-of-concept to determine the causal relationship of SigB deficiency. Indeed, the revertant ID56:*rsbU*(T431G) of the SigB-deficient isolate ID56 displayed increased pigmentation accompanied by increased asp23-transcriptional expression (RT-qPCR), both typical for higher SigB activity (Figure 4A). In addition, exchanging the SNP from G to A at position 368 in *rsbU* in the SigB-functional strains–IN and the human reference strain Newman–induced the SigB-deficient phenotype as shown by abrogating pigment production and *asp23* expression (Figure 4B). Thus, we show in two examples that only a single nucleotide substitution within the phosphatase domain of *rsbU* caused the SigB-deficient phenotype.

## 3. Discussion

In this study, we report a prevalence of 10.4% (n = 77) of bovine mastitis isolates exhibiting the SigB-deficient phenotype. To our knowledge, this is the first time that the prevalence of SigB deficiency among *S. aureus* isolates has been studied. Sequencing of the *sigB* operon (*mazEF*-*rsbUVW*-*sigB*) from each of the eight SigB-deficient isolates revealed several single nucleotide substitutions potentially causing the SigB-deficient phenotype. We identified the phosphatase domain of the RsbU protein as a repeated target of mutations, as two SigB-deficient strains had non-synonymous SNPs in this part of the *rsbU* gene (G395A → S132N; G431T → G144V). Notably, we could confirm the causal relationship of the single SNP in *rsbU*(G431T), as well as the SNP in *rsbU*(G368A) from our previous study demonstrating within-host evolution towards the host-adapted, SigB-deficient variant (HA) [8]. Consequently, a loss in the RsbV-P-specific phosphatase activity of RsbU can be assumed, which is a crucial step in regulating SigB via a partner-switching mechanism, and activation of SigB is abolished. In line with this, we obtained reduced levels of *asp23* expression and pigmentation for both SigB-deficient bovine isolates compared to their isogenic SigB-functional counterpart. Mutations in the *rsbU* gene causing SigB deficiency were known from human staphylococcal infections. Most prominently, the laboratory prototype strain NCTC8325 (RN1) was shown to harbour an 11-base-pair (bp) deletion and the high-protease-producing prototype strain V8 to include the insertion of a 1073 bp IS element in *rsbU* [5,6,7]. Moreover, an 18-bp deletion in *rsbU* was detected in an isolate derived from a chronic cystic fibrosis infection, and a stop codon (TGA) at AA252 was found in the clinical isolate KS26 [5,6,7,28]. Mutations in *rsbU* (IS256 insertion, early stop codon occurrence, substitutions A230T and A276D) were identified after exposing *S. aureus* human clinical isolates to phototoxic conditions (photoantimicrobial chemotherapy-PACT), suggesting a role of the RsbU-dependent SigB activity against reactive oxygen species (ROS) [29]. Our findings from bovine mastitis infections indicate that genetic variation in the *rsbU* gene is important independent of the genetic background or the host. However, in many isolates, we could not identify genetic features within the *sigB* operon that may cause the SigB-deficient phenotype. We assume that the SigB-deficient phenotype may be caused by many different underlying mutations. This requires care when inferring the causative genotype-phenotype relationship at the genetic and (post-)transcriptional level. 

About 14% of the isolates from our mastitis strain collection were non-pigmented. However, studies on the prevalence of non-pigmented *S. aureus* are scarce. A survey conducted in the Shanghai region revealed that 41% (54/132) of isolates were non-pigmented, including clinical and food-related samples [30]. While pigment-producing strains can be considered SigB-functional, reduced pigmentation may be due to SigB deficiency or other factors. For example, knock-out mutations of *crtNM* genes in the STX-producing operon, of the two-gene regulatory operon-*yjbIH* and of genes that are part of the de novo purine biosynthesis pathway have been reported to reduce pigment production [30,31,32,33]. The strong positive correlation between *asp23*-expression (SigB activity) and pigmentation of isolates in our mastitis collection was therefore intriguing and suggested only minor SigB-independent regulatory perturbations on staphylococcal pigmentation. Based on the correlation analysis, it can therefore be assumed that many isolates that produce no amount, or intermediate amounts, of carotenoid pigments (approx. 40%) were likely SigB-dysfunctional or at least SigB-compromised. However, it remains unclear why they do not show all the typical features of the SigB-deficient phenotype.

The co-occurrence of high production of proteases and *a*-hemolysin has been reported for many phylogenetically unrelated SigB-deficient strains [6,8,9]. Given the substantial proteolytic activity observed in the isolates in our bovine mastitis collection, we anticipate the secretion of proteases at a higher level associated with a slower transition towards the expression of toxins (including α-hemolysin). SarA is known to increase the expression of the *agr*/RNAIII system and to repress gene expression of proteases independently of *agr* [11]. SigB directly inhibits expression of the *agr*/RNAIII system but induces expression of *sarA* [24]. The lower SigB activity may help counterbalance the high expression of *agr*/RNAIII and decrease the expression of α-hemolysin in the specific context of these isolates. Moreover, low SarA levels increase the expression and secretion of proteases that were shown to degrade secreted α-hemolysin, possibly contributing to the loss of the α-hemolysin phenotype [34,35]. Furthermore, we recently did not detect secretion of proteases but strong α-hemolysin secretion of a SigB-deficient strain under growth-limiting conditions, indicative of differential regulation of the two virulence factors downstream of the *sigB* operon [10]. One could even speculate that the expression of proteases is evolutionarily favoured over toxins in bovine-adapted *S. aureus*, as milk proteins are readily accessible as a nutritional source in the mammary gland. Nevertheless, regulatory cross-talks might exist between the major regulators *sigB*, *agr*/RNAIII and *sarA*, which fine-tune *S. aureus* gene expression and secretion of proteolytic and cytotoxic virulence factors under certain niche-specific conditions. Thus, repression of SigB could better adapt the pathogen to the extracellular microenvironment. In contrast, SigB activation allows intracellular survival, and the continuous switch between the two lifestyles could then further promote long-lasting, persistent infections.

This work highlights the clinical relevance of SigB deficiency in the pathogenesis of *S. aureus* infections. To our knowledge, this is the first report evaluating the prevalence of SigB deficiency among *S. aureus* isolates. We demonstrate that carotenoid pigmentation correlates with *asp23* expression, underpinning the role of pigmentation as a valuable predictor of the functional status of SigB. Further, we discuss that SigB activity might be fine-tuned rather than completely on or off, allowing an adaptive shift between intracellular and extracellular niches. However, future studies are needed to fully understand the niche-specific adaptation dynamics within the host to clarify the selection pressure acting on SigB during long-lasting, persistent infections.

## 4. Materials and Methods

### 4.1. S. aureus Strains and Growth Conditions

Seventy-seven *S. aureus* isolates were obtained from bovine mastitis milk samples of various geographic origins (Austria, n = 44; Argentina, n = 18; Rwanda, n = 13; others, n = 2) [36,37,38,39,40,41]. The isogenic SigB-functional, initial (IN) and SigB-deficient, host-adapted (HA) bovine isolates were used as a reference for the phenotypic discrimination of pigment production, proteolytic activity, exoproteome pattern analysis and antibody-based detection of a-hemolysin (Hla) (-toxin) secretion [8]. *S. aureus* 6850Δ*hla* (kindly provided by L. Tuchscherr de Hauschopp, Institute of Medical Microbiology, University Hospital of Jena, Germany) and wild-type 6850 [42] were used as assay control strains to test for detection of α-hemolysin secretion. The *rsbU*-repaired SH1000 (SigB-functional) [43], the wild-type 8325-4 (SigB-deficient, 11 bp deletion in *rsbU* gene) [44] and the isogenic mutant SH1000Δ*sigB* (SigB-deficient) [4], as well as strain 6850 (SigB-functional) [42] and its isogenic Δ*sigB* mutant (SigB-deficient) [45], were used as SigB-functional and SigB-deficient reference strains to determine *S. aureus* carotenoid pigmentation. *S. aureus* SH1000 and SH1000Δ*sigB* were included as assay control and reference for mRNA expression studies of *asp23* (RT-qPCR). All *S. aureus* isolates were stored in glycerol stocks at −80 °C until used and were initially grown on tryptic soy agar (TSA) (Thermo Fisher Scientific, Oxoid, Hampshire, UK) at 37 °C, supplemented with erythromycin (5 μg/mL) (Carl Roth, Karlsruhe, Germany) where needed.

### 4.2. Visualization and Determination of Carotenoid Pigmentation

To visualise *S. aureus* carotenoid pigmentation, one millilitre of overnight culture was precipitated by centrifugation (10,000× *g* for 1 min; Eppendorf, Hamburg, Germany) at the bottom of the tube. To quantify *S. aureus* carotenoid pigmentation (incl. STX and intermediate carotenoids), the absorbance of methanol-extracted pigments was measured using an adapted protocol [46]. Overnight cultures were inoculated in 5 mL TSB (Thermo Fisher Scientific, Oxoid, Hampshire, UK) in glass tubes and incubated at 37 °C while shaking at 120 rpm. After 18 h, OD_600_ was measured (BioSpectrometer, Eppendorf, Hamburg, Germany), and samples were normalised to cell mass (8 mL at OD_600_ = 1). Next, cultures were centrifuged at 10,000× *g* for 1 min to harvest the cells. The bacterial cell pellets were resuspended in 800 μL methanol (Carl Roth, Karlsruhe, Germany) and incubated at 55 °C for 3 min to extract the pigments. After removing the cell debris by centrifugation, the supernatant was transferred into new tubes, and 300 μL per sample was added in duplicates to a 96-well plate (Greiner Bio-One, Kremsmünster, Austria). The extracted pigments were quantified by measuring the OD using the SpectraMax M3 (MolecularDevices, San Jose, CA, USA) at an interval of 300 to 750 nm in 1 nm steps. Carotenoid pigmentation was calculated using the area under the curve (AUC) at an OD ranging from 390 to 520 nm, with the baseline adjusted as a line through the OD values at 390 and 520 nm (OriginPro 2022, OriginLab, Northampton, MA, USA) (Supplementary Figure S1). We calculated the cut-off value (AUCc) based on the average mean and standard deviation (SD) of the four SigB-deficient reference strains (SH1000Δ*sigB*, 6850Δ*sigB*, 8325-4 and HA), AUCc = average AUC + 3 x average SD (Supplementary Figure S1). The isolates were further divided into the following categories based on the calculated average SD value of the negative controls: (i) no pigmentation, AUC ≤ AUCc, (ii) intermediate pigmentation, AUC ≤ AUCc + 3 × SD, and (iii) full pigmentation, AUCc + 3 × SD < AUC (adapted from [47]). Each sample was measured in technical duplicates on at least two separate days.

### 4.3. Proteolytic Activity

*Staphylococcus aureus* proteolysis of non- and intermediate-pigmented (n = 30) mastitis isolates was conducted, as reported earlier [8]. Briefly, 1% skim milk broth was prepared using 10 g/L skim milk powder (Thermo Fisher Scientific) in MQ-water (Merck Millipore, Darmstadt, Germany). The solution was autoclaved for 5 min at 121 °C and stored at 4 °C until use. To assess proteolytic activity, 15 μL of a resuspended pellet derived from a 1 mL TSB overnight culture (5000× *g* for 2 min) was added to 3 mL 1% skim milk broth and cultivated at 37 °C without shaking. Results were recorded after 24 h.

### 4.4. α-Hemolysin (Hla) Western Blot and Analysis of Exoproteome Pattern

We tested non- and intermediate-pigmented isolates for increased α-hemolysin secretion and increased protein secretion, both typical phenotypic features of SigB deficiency [9,23]. Sterile filtrated bacterial supernatants were used to determine the amount of secreted α-hemolysin by western blotting and to analyse the bacterial exoproteome pattern. Overnight cultures were inoculated in 3 mL TSB and incubated at 37 °C. After 18 h, the OD_600_ was measured, and the samples were diluted to OD = 0.05/mL in 50 mL TSB and grown in 250 mL Erlenmeyer flasks at 37 °C at 120 rpm to OD_600_ = 5. The cultures were centrifuged at 5000× *g* at 4 °C for 10 min. The supernatant was sterile filtrated using 0.22 μm Millex-filters (Merck Millipore) and kept frozen at −80 °C until use. α-hemolysin western blots were performed according to Mayer et al., 2021 [10], using a combination of the anti-α-hemolysin antibody [8B7]—N-terminal (#ab190467) (Abcam, Cambridge, UK) diluted 1:3000, and the peroxidase-conjugated AffiniPure Goat Anti- Mouse IgG (H + L) antibody (#115-035-062) (Dianova, Hamburg, Germany) diluted 1:20,000. Sodium dodecyl sulfate-polyacrylamide gel electrophoresis (SDS-PAGE) and silver staining were performed to visualise the bacterial exoproteome pattern using a 12% separation gel [48].

### 4.5. Reverse-Transcription (RT)-qPCR

Isolates exhibiting the SigB-deficient phenotype were tested for *asp23*-expression (RT-qPCR) to assess the level of SigB activity [26]. Total RNA extraction and *asp23*-RT-qPCR were performed on bacterial strains grown at 37 °C in TSB (120 rpm and aerobe) according to Marbach et al., 2019 [8] using the indicated primers for *asp23* [4] normalised to the geometric mean of three reference genes (rpoD, rho and dnaN). Relative expression levels were calculated using the REST method [49]. For experiments validating SNPs (Section 2.3.), strains were grown until OD_600_ = 5 in three independent experiments with technical duplicates. For screening SigB activity (Section 2.1.3 and Section 2.2), we used a sample of 24 isolates from our mastitis strain collection, exhibiting all pigmentation phenotypes, including the eight isolates presenting the SigB-deficient phenotype. Strains were grown until OD_600_ = 3 in two independent experiments with technical duplicates. Mean relative expression and standard deviations were calculated from independently grown samples and their technical duplicates.

### 4.6. Genetic Manipulation

Genetic manipulation of *S. aureus* was carried out using the thermosensitive plasmid pIMAY [50] and engineered *E. coli* strains to simplify the transformation of *S. aureus* isolates [51]. For the introduction of *rsbU*(G368A) in various strains, the *rsbU*(G368A) allele was amplified using the chromosomal DNA of the host-adapted isolate (HA) and cloned into pIMAY. The recombinant plasmid was used to transform the initial isolate (IN) and *S. aureus* Newman (NM). Allelic replacement was used to exchange plasmid and chromosomal alleles [50]. Successful replacement was verified by Sanger sequencing. To exchange nucleotide *rsbU*:431T in strain ID57 to *rsbU*:431G, chromosomal DNA of ID57 was used, and two primer pairs (AB and CD) were used to amplify two fragments of *rsbU*. The desired point mutation was integrated into complementary primers B and C. The two fragments were fused by spliced extension overlap PCR (using primers A and D), and the created mutant allele was cloned into pIMAY. The recombinant plasmid was used to transform *S. aureus* ID57, followed by allelic replacement as described above. Oligonucleotides used for genetic manipulation in this study were summarized in Supplementary Table S1.

### 4.7. Sequencing of the SigB-Locus and Molecular Strain Typing

The *sigB*-locus (*mazEF-rsbUVW*-*sigB*) was amplified using the following primers: SigB-A_for, GCAGTGTTAATACTGCTTC and SigB-B_rev, GTTAATGAAGGAACGGAGG, [52]; rsbU_for, GAGAAATACACTGACGAAG [53] and rsbU_rev, CACAGCTTCACTAACTGCAA [53] and rsbU_RS_for, ACAGCAAAGCTCATTGTGCC (this study) and rsbU_RS_rev, AGGGAAGTGAGGAGGCAACT (this study); mazEF_for, AACCAAAGCCTTTAACGATTTCT (this study) and mazEF_rev, AGACGTTTGCCGCGAATCTA (this study). The following cycling conditions were used: initial denaturation at 98 °C/30 s, followed by 30 cycles of denaturation at 98 °C/10 s, annealing at 54 °C (*sigB*), 57 °C (*rsbU*), 59 °C (*mazEF*) and 60 °C (*rsbU_RS*) for 30 s and extension at 72 °C/60 s followed by a final extension at 72 °C/7 min. Genomic information of known SigB-functional strains was retrieved from the NCBI website (SH1000, 6850, COL, Newman, USA300_FPR3757, RF122) or in-house sequencing (IN) and used to analyse the *sigB*-locus. Selected isolates were genotyped using MLST as previously described [37].

### 4.8. Statistics

Differences in relative *asp23* expression were tested by an unpaired Student *t*-test (two-tailed) of log-transformed data. Minimum statistical significance was set to *p* < 0.05. A correlation analysis (Pearson r, correlation *p*-value) was performed on log-transformed relative *asp23*-expression levels and the area under the curve (AUC) of carotenoid pigmentation. GraphPad Prism software (San Diego, CA, USA) version 7.0 was used for all statistical comparisons and visualisations.

## Figures and Tables

**Table 1:** Characteristics of isolates presenting the SigB-deficient phenotype.

Isolate	Phenotypic Traits of SigB-deficiency				SigB-activity	*sigB*-operon	ST (CC)
ID	Pigmentation	Proteolysis	Hla	Protein	rel. *asp23*	Mutations compared to	
	(AUC)		secretion	secretion	expression	SigB-functional strains	
**17**	−0.141	+	+	+	176.05	no difference	ST504 (151)
**18**	−0.411	+	+	+	43.26	syn. SNPs in *mazeF*/*rsbW*/*sigB* and one SNP in the non-coding region between *rsbV* and *rsbU*	ST5477 (3666)
**31**	−0.390	+	+		23.55	SNP *rsbU*(G395A → S132N) ^#^	ST8 (8)
**37**	0.291	+	+	+	76.30	no difference	ST504 (151)
**48**	0.447	+	+	+	76.02	no difference	ST151 (151)
**54**	0.386	+	+	+	19.89	no difference	ST97 (97)
**56**	−1.150	+	+		0.99	SNP *rsbU*(G431T → G144V) *^##^*	ST9 (9)
**67**	−0.980		+	+	142.00	no difference	ST5476 (3591)
Mastitis-derived SigB-deficient reference strain (Marbach et al., 2019)							
**HA**	−1.10	+	+	+	4.83	SNP *rsbU*(G368A → G122D) *^##^*	ST352 (97)

# putative, ## confirmed SNP causing SigB-def.

## Data Availability

The authors confirm that the data supporting the findings of this study are available within the article and its Supplementary Materials.

## Supplementary Materials

The following supporting information can be downloaded at: https://www.mdpi.com/article/10.3390/antibiotics12040699/s1, Supplementary Figure S1, Determination of carotenoid pigmentation; Supplementary Figure S2, *asp23*-mRNA expression of mastitis isolates; Supplementary Table S1, Oligonucleotides used for genetic manipulation in this study.
