# Supplementary material for: Prevalence of the SigB-Deficient Phenotype among Clinical Staphylococcus aureus Isolates Linked to Bovine Mastitis
Source: Antibiotics (Basel). 2023 Apr 3;12(4):699. doi: 10.3390/antibiotics12040699 (PMC10135042; doi:10.3390/antibiotics12040699)
Supplement: Supplementary file 1 [file antibiotics-12-00699-s001.zip › antibiotics-2284912-supplementary.pdf]

Figure S1, Determination of carotenoid pigmentation

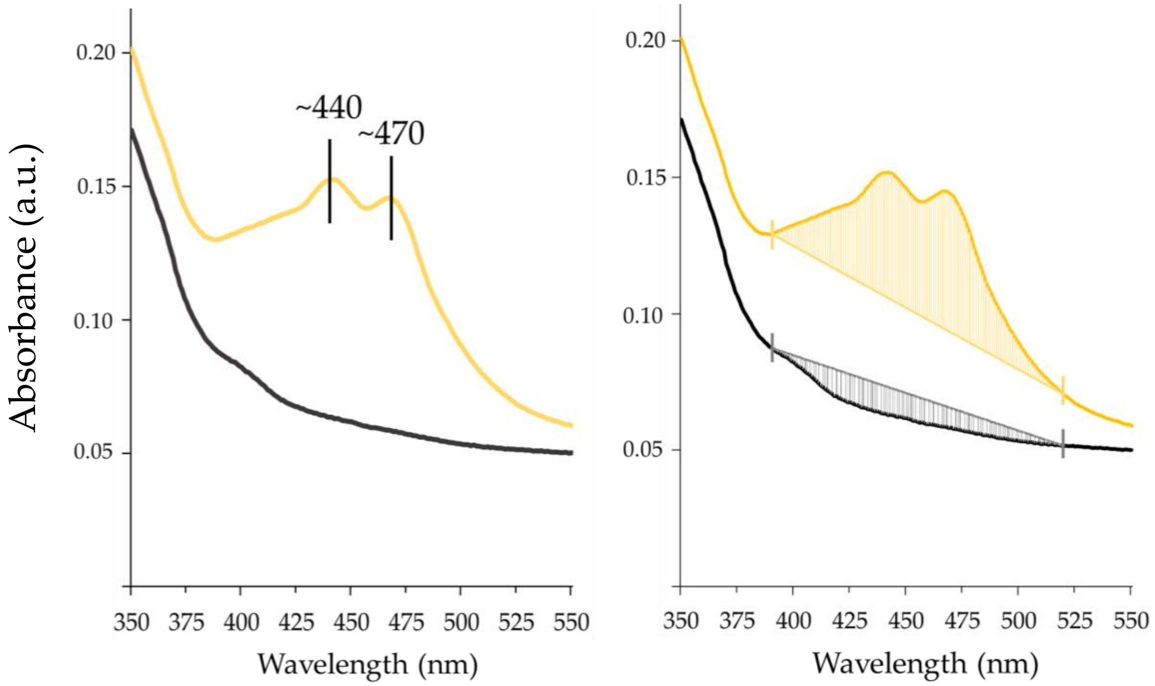

Average spectra and AUC of SigB-functional (SH1000, 6850, and IN) and SigB-deficient (SH1000Δ*sigB*, 8325-4, 6850Δ*sigB* and HA) reference strains. Spectral profile and characteristic peak maxima at ~ 440 nm and ~ 470 nm of *S. aureus* carotenoid pigmentation; Area under the curve (AUC) for each spectrum with baseline adjusted as a straight line through the OD values at 390 and 520 nm. AUC of SigB-functional strains (yellow hatched) and AUC of SigB-deficient strains (black hatched). a.u., arbitrary units.

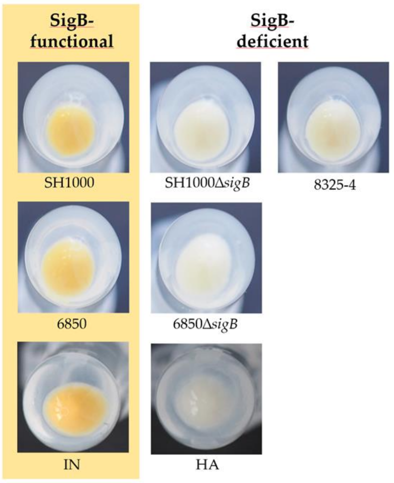

Reference strains		SigB-activity	$AUC_{390-520}$	$AUC_{390-520}$
SH1000		+	3.8827	
	SH1000Δ*sigB*	-		-0.7956
	8325-4	-		-0.6586
6850		+	2.5503	
	6850Δ*sigB*	-		-0.8975
IN		+	2.7089	
	HA	-		-1.1001
average $AUC_{390-520}$ of SigB-deficient reference strains				-0.8630
SD ($AUC_{390-520}$) of SigB-deficient reference strains				0.2194

Bacterial pigmentation (centrifuged pellet) and measured $AUC_{390-520}$ values of extracted pigments of SigB-functional and -deficient reference strains.

Figure S2, *Asp23*-mRNA expression of mastitis isolates

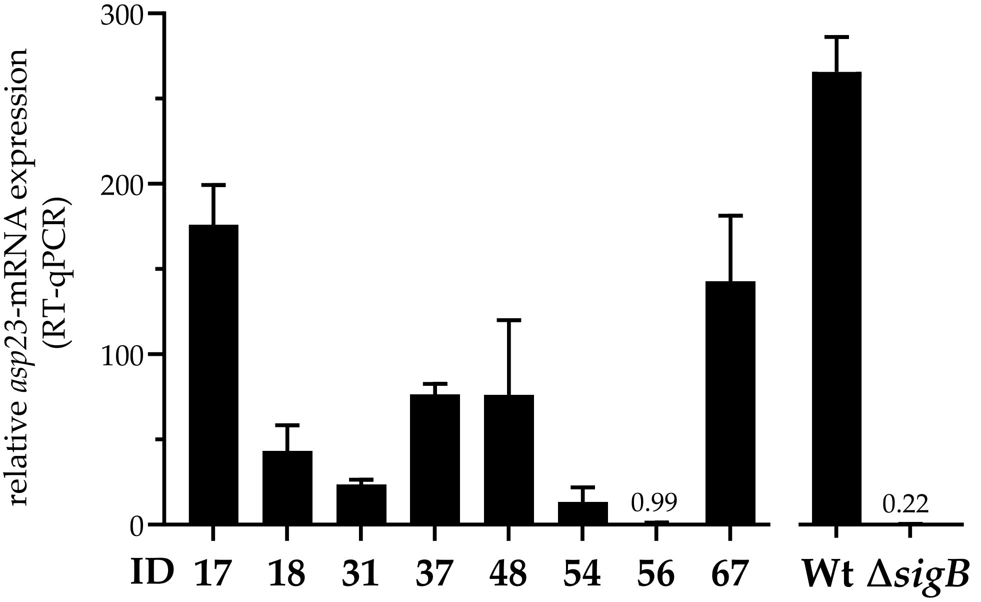

Asp23-mRNA expression (RT-qPCR) of mastitis isolates exhibiting the SigB-deficient phenotype. SH1000 (WT) and the isogenic Δ*sigB* mutant were included as assay control and reference.

Table S1, Oligonucleotides used for genetic manipulation in this study

**Name**	**5′-3′ sequence**	**Restriction site**	**Ref.**
rsbU:G368A_F	TATAGTCGACTTTAAGCGCTGTATCCACCA	SalI	this study
rsbU:G368A_R	TATAGAGCTCACGCCTAATTTTTCTGGTGAGC	SacI	
rsbU:T431G_A	AGGGAACAAAAGCTGGGTACCAGGGTTTAATTGATGAAAGTTTAAC	KpnI	
rsbU:T431G_B	GCAAAGCTCATTGTG**c**CATCGTTATGG *		
rsbU:T431G_C	CCATAACGATG**g**CACAATGAGCTTTGC *		
rsbU:T431G_D	AACATAAACATATGCACCCACAAGGAGCTCCAATTCGCCCTAT	SacI	

* Targeted point mutations to introduce the T431G exchange are indicated by lower case letters
